# Electroencephalography Based Fusion Two-Dimensional (2D)-Convolution Neural Networks (CNN) Model for Emotion Recognition System

**DOI:** 10.3390/s18051383

**Published:** 2018-04-30

**Authors:** Yea-Hoon Kwon, Sae-Byuk Shin, Shin-Dug Kim

**Affiliations:** Department of Computer Science, Yonsei University, 50 Yonsei-ro, Seodaemun-gu, Seoul 03722, Korea; yeahoon.kwon@yonsei.ac.kr (Y.-H.K.); dawnshin2000@yonsei.ac.kr (S.-B.S.)

**Keywords:** EEG, GSR, emotion recognition, deep learning, convolution neural networks, pattern recognition, hybrid neural network

## Abstract

The purpose of this study is to improve human emotional classification accuracy using a convolution neural networks (CNN) model and to suggest an overall method to classify emotion based on multimodal data. We improved classification performance by combining electroencephalogram (EEG) and galvanic skin response (GSR) signals. GSR signals are preprocessed using by the zero-crossing rate. Sufficient EEG feature extraction can be obtained through CNN. Therefore, we propose a suitable CNN model for feature extraction by tuning hyper parameters in convolution filters. The EEG signal is preprocessed prior to convolution by a wavelet transform while considering time and frequency simultaneously. We use a database for emotion analysis using the physiological signals open dataset to verify the proposed process, achieving 73.4% accuracy, showing significant performance improvement over the current best practice models.

## 1. Introduction

Multimodal human and computer interaction (HCI) has been actively researched over the last few years. One outstanding issue is affective computing, designing devices that communicate with humans by interpreting emotions. Emotion recognition has been attracting attention as a next-generation technology in many fields, from the development of humanistic robots to consumer analysis and safe driving. Most previous research has classified emotions using only facial expressions. However, facial expressions only represent part of the overall human emotional response, and emotion discriminators can sometimes make significant mistakes. For example, classifying an athlete's image as displaying happy emotion, when actually the smiling athlete is nervous prior to an important game [1]. On the other hand, biological signals from the central (CNS) and the peripheral (PNS) nervous systems are hard for humans to mentally control, and can accurately represent emotions. Previous studies have shown that changes in skin signals (i.e., galvanic skin response (GSR)) are closely related to changes in peripheral nerves with emotional changes [2], and electroencephalogram (EEG) signals from the frontal lobe are strongly related to emotional changes [3,4]. Therefore, the current study classified emotions using biological signals, including EEG and GSR.

Electroencephalogram signals are used in brain computer interface research, measuring brain electrical activity using an electrode that is attached to the scalp. However, reduced accuracy due EEG signal instability remains a major problem, and EEG signals are untrustworthy, even when they are employing expensive and reliable equipment. The solution is to use as many heterogeneous sensors as possible to provide reliable multiple data. Therefore, we designed a data adaptive CNN model to improve the emotion classification accuracy, reducing current model instabilities, using EEG and GSR data. We also implemented effective spectrogram feature extraction and designed a multimodal classifier that takes two features as input at the first layer of a fully connected network.

This paper is organized as follows. Section 2 discusses previous research methodologies and results. Section 3 discusses the current paper’s main contributions, including the details of label processing, EEG signal transformation, GSR data feature extraction, and introduces the proposed CNN model architecture and training strategy. Section 4 analyzes the results and compares them with the current best practice models. Finally, Section 5 summarizes and concludes the paper.

## 2. Related Work

The related research fields of emotion classification and EEG preprocessing have achieved remarkable results. In general, preprocessing EEG data consists of selecting data while considering the frequency and the location of the brain. Fast Fourier transform (FFT) is the most common frequency analysis method for raw EEG data [5,6,7,8,9], and it was adopted here to extract EEG features. However, FFT cannot reflect temporal information in the frequency data, requiring additional methods to recognize emotions over time. Therefore, short time Fourier transform (STFT), which can express frequency per hour [10,11,12,13], was also used to analyze EEG signals.

**Figure 1:**
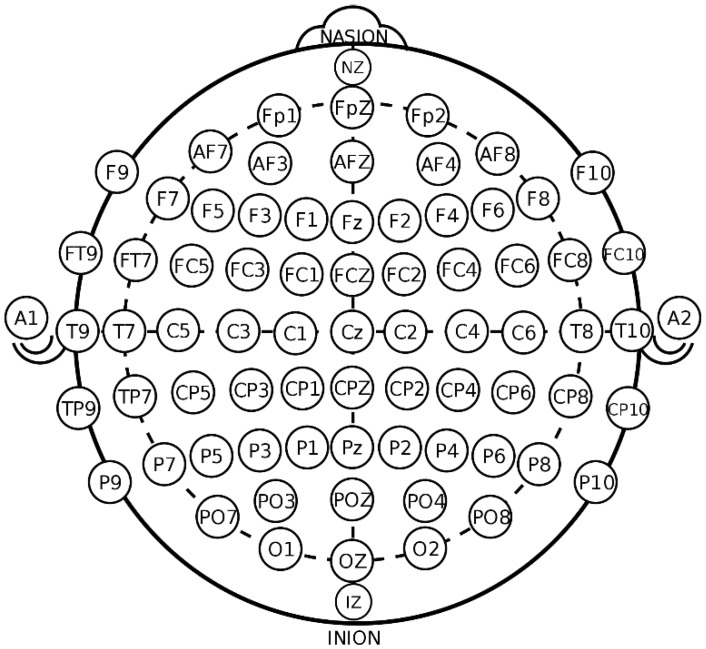
The 10-20 system the international standard and location of the electrodes.

Classifying EEG features by frequency is the most common method to differentiate alpha, beta, theta, and gamma waves. Liu et al. [14] presented a table of emotions by frequency and electrode location within the brain region. Figure 1 shows the location of the electrodes that are attached to the scalp using the 10-20 system, which is the international standard. Electrodes F3 and F4 distinguish between negative and positive emotional states, and AF3 andAF4 distinguish positive emotions from the surrounding emotions. Wavelet analysis is one of the best ways to express frequency and time, and has also been employed in EEG classification [15,16,17,18].

Various previous studies considered emotional classification methods. Mollahosseini et al. [19] designed a CNN based face recognition module. Gerard Pons et al. [20] enhanced facial image classification performance by supervised hierarchical learning. Ding et al. [21] performed deep face recognition that was based on a two steps model. Poria et al. [22] implemented multimodal visual and audio data analysis beyond the focus on text-based emotional analysis. They also succeeded in feature fusion through deep learning based heterogeneous data dimension reduction.

The Database for Emotion Analysis using Physiological signals (DEAP) dataset has been widely employed for emotion classification models using biomedical signals. Koelstra et al. [23] used the DEAP data set to classify PNS and CNS sensor data, and measured the emotional classification performance. Liu and Sourina [24] studied EEG valence levels for real-time applications. Naser et al. [25] predicted emotions extracted from music videos. Chen et al. [26] applied ontology and datamining techniques for EEG based emotion analysis.

Bayesian networks, unsupervised deep running, and deep belief networks have also been applied [27,28,29].

## 3. Methods

### 3.1. Multiple Label Classification

**Figure 2:**
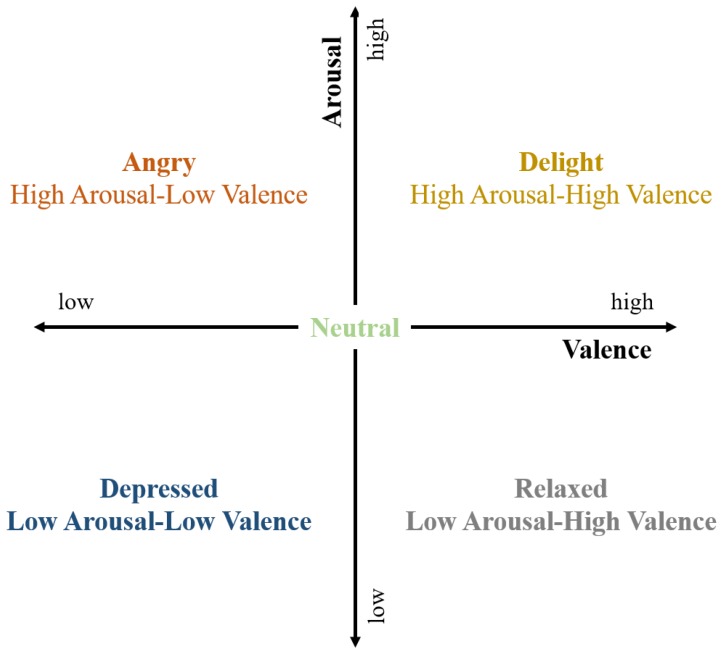
Arousal, valence two-dimension plane.

A label was constructed using the self-assessment value that was provided in the DEAP dataset, including valence, arousal, dominance, liking, and familiarity. Emotional states are typically evaluated using arousal and valence, and are divided into four sections: high arousal, high valence (HAHV); high arousal, low valence (HALV); low arousal, low valence (LALV); and, low arousal, high valence (LAHV) [30], as shown in Figure 2. Thus, emotional states can be classified according to arousal and valence levels.

Labeling was based on a threshold value for the two-dimensional (2D) plane. We implemented k-means clustering on self-assessed arousal and valence levels to find the most appropriate threshold. Previous studies have employed one shot encoding for labeling as a 2D vector, i.e., [HV, LV] and [HA, LA] using k-means clustering with k = 2 [31]. Therefore, we performed independent valence and arousal classifications in order to compare with previous models.

However, independent classification fails to consider arousal and valence correlations, and since the data is arousal and valence levels, rather than emotion level, it cannot be implemented for end to end learning, since it must be mapped onto the two-dimensional (2D) plane (Figure 2) for emotion judgment.

**Figure 3:**
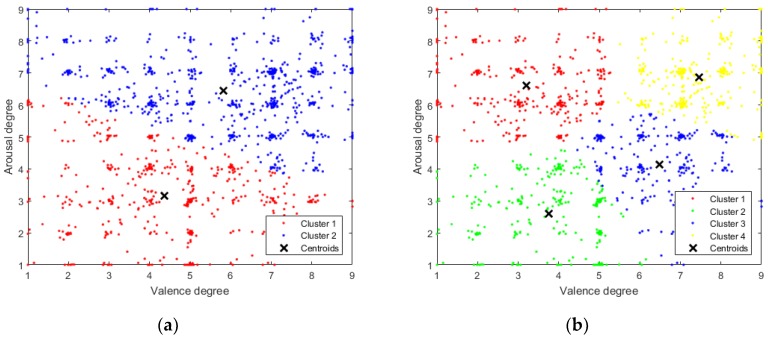
K-means clustering results of Arousal-Valence self-assessment data (**a**) Clustered Arousal-Valence data result when k = 2; and, (**b**) Clustered Arousal-Valence data result when k = 4.

Therefore, we propose k-means clustering with k = 4 to provide a four-dimensional (4D) label vector. Figure 3 compares clustering for k = 2 and k = 4. Point (5, 5) is the approximate center mean for both k = 2 and k = 4, hence we use (5, 5) as the threshold.

Thus, labeling included 2D and 4D vectors through one shot encoding for learning.

### 3.2. EEG Signal Transformation to Time to Frequency Axes

The data was preprocessed to reflect EEG temporal and frequency characteristics. Since the EEG data measuring human emotions are time series data, time information must be reflected in the frequency data. Although the STFT has been widely used to add time information to frequency data [10,11,12,13], it has disadvantages for time-frequency analysis, in that temporal resolution decreases as the window increases; and, frequency resolution decreases as window size decreases.

**Figure 4:**
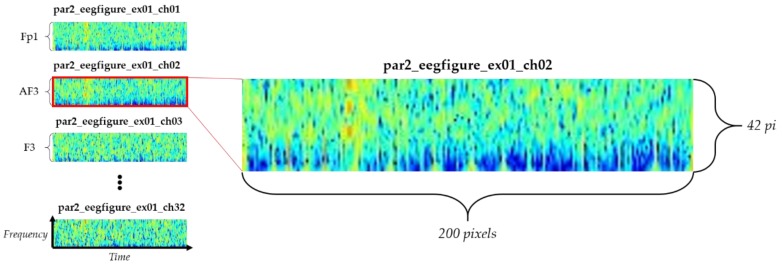
Wavelet transformed spectrogram for each electrode.

Therefore, we propose using a wavelet transform to represent the frequency axis, using the open toolbox EEG lab. The extracted spectrogram was 42 × 200 pixel, width × height, where width (200 pixel) represents time, and height (42 pixel) represents EEG sensor frequency (4.0–45 Hz), as shown in Figure 4. Total transformed data include 40960 spectrograms. At this time, the number of batch data for training is 32 spectrogram data that means 32 electrodes that were derived from one stimulus. Therefore, the total amount of data set used in this study is 1280, with data labels, as shown in Table 1 and Table 2.

Conventional EEG based emotion classification analyzes the degree of activity in a specific area of the brain (e.g. the frontal lobe), using electrodes that were attached to the head close to the frontal lobe and some other lobes (e.g., AF3, AF4, P7). Frequency bands for specific electrodes were typically subdivided into alpha, beta wave, gamma, etc. waves to allow for simple and shallow classification models, such as support vector machines (SVMs). However, sensor selection and subdivision ignores emotion related signal changes in other brain regions. Recent advanced deep learning techniques can improve emotional analysis accuracy by incorporating all sensor data for each experiment.

### 3.3. GSR Preprocessing Using Short Time Zero Crossing Rate

To extract the feature, we divide the GSR waveform into defined windows and calculate the short time zero crossing rate (STZCR), i.e., the number of times the signal crosses zero within a given window. That is, we intend to use the change in amplitude of the GSR as the input feature vector for deep running. STZCR indicates the rate of signal change,

(1) $$STZCR=\frac{1}{N}\overset{m}{\underset{n}{\sum }}\frac{\left|sig\left\{s\left(n\right)\right\}-sig\left\{s\left(n-1\right)\right\}\right|}{2}w\left(m-n\right)$$

where N is the sampled signal, and w represents the window. We highlighted features using the extracted zero crossing rate vector with threshold

(2) $$T=\frac{\sum GSR_{stzcr}}{N_{stzcr}}$$

where $GSR_{stzcr}$ is a vector column and $N_{stzcr}$ is the number of vectors. If the data is greater than the threshold, it outputs max, otherwise it outputs zero. GSR amplitude is generally sensitive to arousal changes and less sensitive to valence changes, hence, it can positively affect EEG features to focus on arousal in the classifier model.

### 3.4. Fusion Convolution Neural Network Model for EEG Spectrograms and GSR Features

Many neural networks have been developed for classification in recent studies. The first thing to consider when designing a CNN is data characteristics. Therefore, we designed the CNN to use the spectrogram image from the wavelet transformation of all the channels.

Tabar and Halici [32] considered CNN classification problems using EEG spectrograms, and designed a single layer CNN using one-dimensional filtering to provide good classification performance based on motor imagery EEG signals. However, a single filtering through the single convolutional layer does not efficiently extract features for emotion classification, since it is not deep enough to extract emotion data.

**Figure 5:**
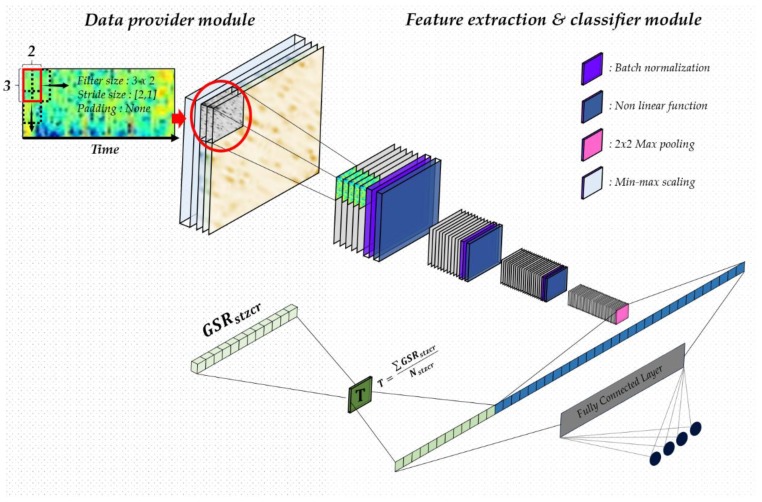
Proposed convolution neural network combining electroencephalogram (EEG) and wavelet transformed galvanic skin response (GSR).

Therefore, we propose a neural network based on the extracted data as described above, which allows for deep convolution layers, while also reflecting temporal effects, as shown in Figure 5. We first normalized the data, making the cost function a spherical contour, and helping to increase the learning rate. We then designed a deep convolution layer that reflects time, using a 3 × 2 filter rather than conventional square filters, such as 2 × 2 or 3 × 3. The spectrogram frequency per hour can be reflected by increasing the filter height. Since the filter is a feature identifier that extracts the information from the manifold state, the shape of the filter is related to the content of the feature to be extracted from the receptive field. Our proposed filter can identify data in a region that is relatively longer than a square filter. Thus, the data containing the vertical meaning is repeatedly transmitted to the input of the next layer. As a result, the frequency per hour of the spectrogram image can be learned in CNN. Setting stride = [2, 1] with no padding, the filter can be extracted based only on the image time base. We used a fully connected layer for the final classification. The classifier is trained on the spectrogram features of 32 electrodes extracted through CNN. In continuous training, the classifier learns similar patterns extracted from 32 individual electrodes, and can be classified as a label through the last softmax layer. The entire model consists of four convolutional layers and seven fully connected layers.

Batch normalization [33] was performed before each value was passed to the activation function, except for the last convolutional layer, in order to prevent the model gradient vanishing during training. It has the effect of preventing internal covariance shift by reducing activation function variation that is caused by the previous layer’s variation. Batch normalization was implemented, as follows.

(1) Normalize the batch data using the batch mean, $\mu_{\beta }$, and variance, $\sigma_{\beta }^{2}$,
  (3) $${\hat{x}}_{i}=\frac{x_{i}-\mu_{\beta }}{\sqrt{\sigma_{\beta }^{2}+\epsilon }}$$
(2) Use the r and d values for scale and shift operations,
  (4) $$y_{i}=\gamma {\hat{x}}_{i}+\beta$$

Updating γ and β by training allows for the CNN to better reflect the model characteristics model in normalized variables, rather than simple normalization, such as whitening. Testing uses average γ and β obtained.

Feature maps are generated as the image passes through each convolution layer. The layer activation function is a rectified linear unit (ReLU), which is a function that makes the value of the part where *x* < 0 in the linear function *y* = *x* is 0,

(5) $$ReLU\left(u\right)=max\left(u,0\right),\;max\left(u,0\right)=\{\begin{array}{ll} u, & if\;u>0\\ 0, & otherwise \end{array}$$

The ReLU function is computationally efficient because its activation is not restricted to [−1, 1], as for the hyperbolic tangent function, but is used as it is. Therefore, training speed for large spectrogram images is increased, and outputting 0 prevents overfitting due to training many weights, hence training regularization can be expected.

After passing through the final 2 × 2 pooling layer, the image is flattened and combined with GSR. To positively influence EEG data performance classification, GSR data uses the data average as the thresh hold to remove noise. It also reduces the computation burden for training a fully connected network by transmitting a zero value to each neuron’s perceptron. The final layer neuron returns the model’s probability distribution using softmax, and performs classification by changing the number of neurons according to the experimental environment, such as [HV, LV], [HA, LA], or [HVHA, LVHA, LVLA, HVAL].

### 3.5. Training Strategy

We use maximum likelihood estimation (MLE) in order to train the proposed CNN model. MLE maximizes P(Y|X;θ) by optimizing *θ* in the probability model for a given data point X and label Y. Cross entropy is the most commonly used MLE loss function, and it calculates the difference between two probability distributions. Let p(x) be the actual and q(x) be the predicted probability distribution for the label. Then, cross entropy, L(p,q), is

(6) L(p,q)=∫p(x)·lnq(x)dx

CNN training proceeds by back propagation using the gradient decent. We update the weights using the partial derivative of cross entropy loss L for weight matrix W,

(7) $$W=W-\gamma \cdot \frac{\partial L}{\partial W}$$

where $z_{j}=\sum w_{ij}o_{i}+b$ is the sum of inner products, and we calculate the gradient as

(8) $$\frac{\partial L}{\partial W}=\frac{\partial L}{\partial p\left(z_{j}\right)}\cdot \frac{\partial p\left(z_{j}\right)}{\partial z_{j}}\cdot \frac{\partial z_{j}}{\partial w_{ij}}$$

where $\frac{\partial L}{\partial p\left(z_{j}\right)}$ is the magnitude of the influence of function p on L and $p\left(z_{j}\right)$ is the softmax result.

**Figure 6:**
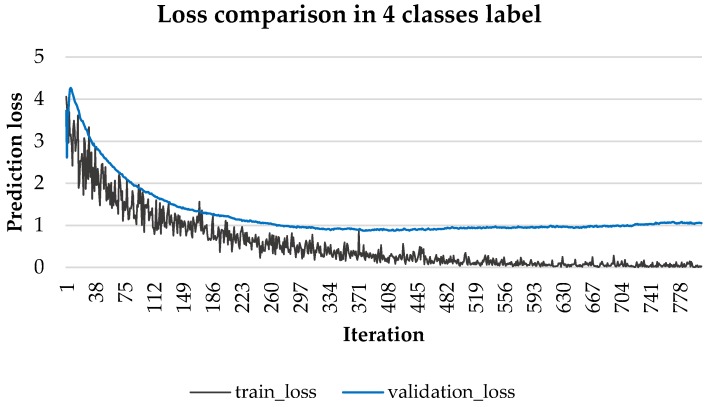
Four class loss to find the optimal training point.

Generally, to find the optimal training point, we find the bias variance trade off point using validation loss, as shown in Figure 6 for the 4 class case.

After 400 iterations, validation loss increases, whereas training continues to decrease. Thus, we can conclude the model becomes over-fitted beyond 400 iterations, providing the optimal training point. Test data should be applied with this level of iteration to measure model accuracy.

## 4. Results and Discussions

### 4.1. Experiment Environment

Table 3 shows the hardware and framework specifications for the experiment.

### 4.2. Dataset

The DEAP dataset [23] was used to provide bio-signal data, containing CNS and PNS data. PNS data comprised GSR, skin temperature, respiration, blood volume (by plethysmograph), and electrooculogram (EOG). GSR data was the skin resistance of the middle and forefinger, skin temperature, and breath change by emotion, including body tension and irritating fear. Plethysmograph measured blood flow changes in the finger. EOG signal was measured by eye blinking, which is related to anxiety. CNS data was the EEG signal.

Data were collected from 32 subjects for 1 m for each of 40 selected music videos. Data was recorded on 48 channels with 512 Hz sampling frequency. We used preprocessed data version of MATLAB and numpy formats that were provided by the DEAP dataset, down-sampled to 128 Hz with a 4.0–45 Hz band pass filter applied.

### 4.3. Performance Analysis

In this section, we analyzed the performance of the model in two ways. In first evaluation, we analyzed the classification performance for each label using hold-out validation. To construct a hold-out validation set, test, verification, and learning datasets were created 1:1:9 ratio for each label, with batch size = 32 to reflect data from one stimulus. Second, for the LOOCV, we constructed the dataset that was measured by one-video as test set and the other video data as training set. The DEAP dataset consists of a data set for 40 videos per participants. In other words, the second evaluation was performed with 39 video stimuli as training dataset, and the data that was extracted by the other one stimulus was used as a test dataset.

The desired ideal model would accurately distinguish data patterns and generalize them even when testing data are considered, i.e., we want to find a model between over and under fitting. The proposed model does not apply L2 regularization to prevent overfitting, because there is a batch normalization layer. In addition, cross entropy loss was measured for each iteration to find the optimal training point, as shown in Figure 6. Table 4 shows the predicted accuracy for methods of label based and video based classification using each validation method.

### 4.4. Comparison with Existing Models

We used two class labels that were commonly adopted in previously studies to compare performance, as measured by arousal and valence classification accuracy for the DEAP dataset. Table 5 shows the performance compared with the existing models measured using the same dataset. The performance of our model is shown by the result of LOOCV in Section 4.3, to validate the generalized performance of the model.

**Figure 7:**
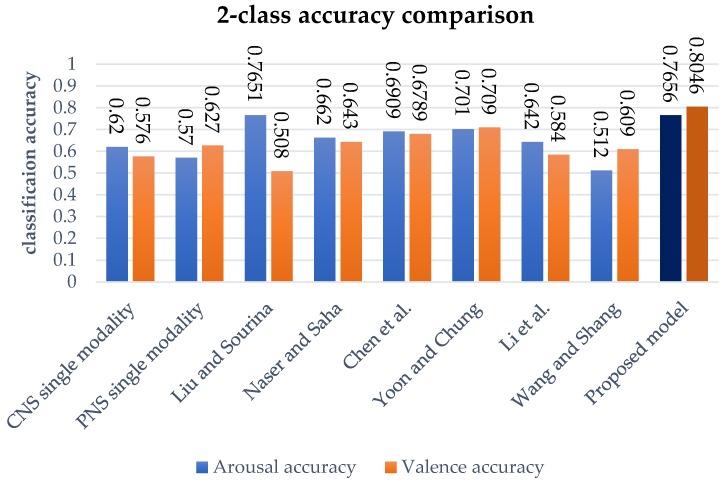
Arousal and valence classification accuracy.

The considered methods used a variety of approaches: Koelstra et al. [23] used CNS and PNS sensors; Liu and Sourina [24] used a fractal algorithm to reflect signal complexity that was based on a threshold value; Naser and Saha [25] extracted features using a dual-tree complex wavelet transform and used SVM for classification; Chen et al. [26] used decision trees; Yoon and Chung [27] used Bayesian and perceptron convergence; and, Wang et al. [28] and Li et al. [29] used deep belief networks to automatically extract features and to classify them. Figure 7 show the proposed model has better performance than all compared models

Although EEG data is easier to classify into two classes [34], increasing the number of classes not only enables end-to-end learning, but it also includes correlations between arousal and valence. Therefore, we compared the proposed model performance against previous four class models. Generally, when data quantity is limited, the model accuracy decreases as the number of labels to be classified increases. The performance of our model is shown by the result of LOOCV in Section 4.3, in order to validate the generalized performance of the model. Table 6 shows that the proposed model has high performance when compared to current models.

**Figure 8:**
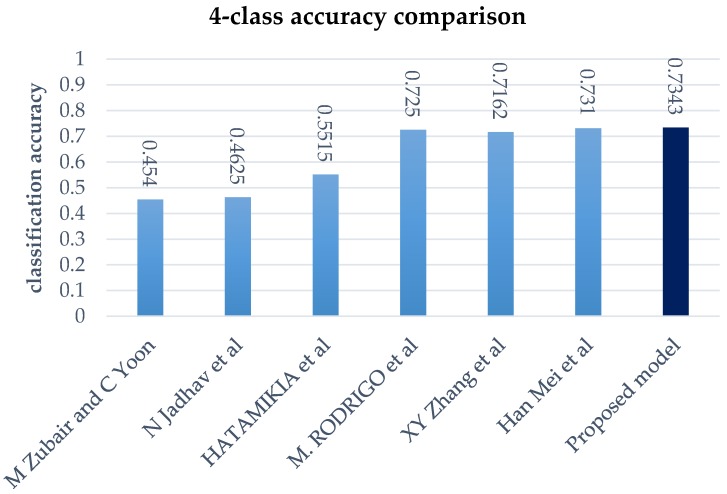
Four class classification accuracy.

A variety of approaches were employed in the comparison models: Zubair and Yoon [35] used a discrete wavelet transform, and also applied the mRMR algorithm to enhance the feature correlations; Jadhav et al. [36] extracted EEG features using the gray level co-occurrence matrix, and classified emotion using K-nearest neighbor; Hatamikia et al. [37] used using nonlinear extraction and self-organized classification; Martínez-Rodrigo et al. [38] extracted biological signal features using quadratic sample entropy, performed feature selection, and classified the extracted features by SVM; Zhang et al. [39] used wavelet feature extraction that was based on a smoothed pseudo Winger-Ville distribution and classification using SVM; Mei et al. [40] extracted features by constructing a connection matrix of the brain structure, with subsequent classification using CNN. Figure 8 show a bar graph that the proposed model has high performance when compared to the current models.

## 5. Conclusions

This study devised data labeling according to emotion criteria, and proposed a data preprocessing methodology to increase the emotional classification performance. Emotion classification was performed using single and multiple sensor based models. Particular focus was overall analysis and CNN filter design according to input data characteristics and noise removal for data processing.

Feature extraction performance was remarkably improved through the proposed filter design, providing significantly improved classification performance when compared with previous models.

This study paves the way for combining data and designing corresponding deep running models. Future research directions will investigate further changes to the emotion analysis framework, such combining multiple neural networks. One approach would be to improve concatenation of simple convolution layers. It may be possible to construct convolution layers for each data characteristic and improve the classification performance using multiple convolution layers.

## Figures and Tables

**Table 1:** The number of extracted wavelet transformed data for two types of labels.

Label ^1^	Data quantity
HAHV	458
HALV	294
LAHV	255
LALV	273
Total	1280

^1^ H: high, L: low, V: valence, A: arousal, e.g., HAHV: high arousal, high valence.

**Table 2:** The number of extracted wavelet transformed data for four types of labels.

Label ^1^	Data quantity	Label ^2^	Data quantity
HA	752	HV	713
LA	528	LV	567
Total	1280	Total	1280

^1, 2^ H: high, L: low, V: valence, A: arousal, e.g., HA: high arousal.

**Table 3:** Hardware specifications.

**CPU**	Intel Core i5-6600
**GPU**	NVIDIA GeForce GTX 1070 8GBytes
**RAM**	DDR4 16GBytes
**OS**	Ubuntu 16.04.
**Frameworks**	Tensorflow1.3 MATLAB/ EEG toolbox

**Table 4:** Emotion classification accuracy.

	Results A ^1^			Results B ^2^		
**Clssification Methods**	Two Class Classification Accuracy ^3^		Four Class Classification Accuracy ^4^	Two Class Classification Accuracy ^3^		Four Class Classification Accuracy ^4^
	Arousal	Valence		Arousal	Valence	
**Proposed Fusion Model**	0.7812	0.8125	0.7500	0.7656	0.8046	0.7343

^1^ Results A: label based classification using hold-out validation.

^2^ Results B: video based classification using leave-out one cross validation.

^3^ Arousal: HA, LA; Valence: HV, LV.

^4^ HAHV, HALV, LALV, LAHV.

**Table 5:** Two class classification performance.

Model		Accuracy	
		Arousal	Valence
CNS feature based single modality	[23]	0.6200	0.5760
PNS feature based single modality	[23]	0.5700	0.6270
Liu and Sourina	[24]	0.7651	0.5080
Naser and Saha	[25]	0.6620	0.6430
Chen et al.	[26]	0.6909	0.6789
Yoon and Chung	[27]	0.7010	0.7090
Li et al.	[29]	0.6420	0.5840
Wang and Shang	[28]	0.5120	0.6090
Proposed fusion CNN model		**0.7656**	**0.8046**

**Table 6:** Four class classification performance.

Model		Accuracy
M Zubair and C Yoon	[35]	0.4540
N Jadhav et al.	[36]	0.4625
Hatamikia et al.	[37]	0.5515
Martínez-Rodrigo et al.	[38]	0.7250
Zhang et al.	[39]	0.7162
Mei et al.	[40]	0.7310
Proposed fusion CNN model		**0.7343**
